# Trainee Autonomy and Supervision in the Modern Clinical Learning Environment: A Mixed-Methods Study of Faculty and Trainee Perspectives

**DOI:** 10.21203/rs.3.rs-2982838/v1

**Published:** 2023-06-06

**Authors:** Stephanie M. Conner, Nancy Choi, Jessica Fuller, Sneha Daya, Peter Barish, Stephanie Rennke, James D. Harrison, Sirisha Narayana

**Affiliations:** Washington University School of Medicine; University of California-San Francisco; University of North Carolina – Chapel Hill; Medstar Georgetown University Hospital; University of California-San Francisco; University of California-San Francisco; University of California-San Francisco; University of California-San Francisco

**Keywords:** Trainee autonomy, supervision, clinical learning environment

## Abstract

**Background:**

Balancing autonomy and supervision during medical residency is important for trainee development while ensuring patient safety. In the modern clinical learning environment, tension exists when this balance is skewed. This study aimed to understand the current and ideal states of autonomy and supervision, then describe the factors that contribute to imbalance from both trainee and attending perspectives.

**Methods:**

A mixed-methods design included surveys and focus groups of trainees and attendings at three institutionally affiliated hospitals between May 2019-June 2020. Survey responses were compared using chi-square tests or Fisher’s exact tests. Open-ended survey and focus group questions were analyzed using thematic analysis.

**Results:**

Surveys were sent to 182 trainees and 208 attendings; 76 trainees (42%) and 101 attendings (49%) completed the survey. Fourteen trainees (8%) and 32 attendings (32%) participated in focus groups. Trainees perceived the current culture to be significantly more autonomous than attendings; both groups described an “ideal” culture as more autonomous than the current state. Focus group analysis revealed five core contributors to the balance of autonomy and supervision: attending-, trainee-, patient-, interpersonal-, and institutional-related factors. These factors were found to be dynamic and interactive with each other. Additionally, we identified a cultural shift in how the modern inpatient environment is impacted by increased hospitalist attending supervision and emphasis on patient safety and health system improvement initiatives.

**Conclusions:**

Trainees and attendings agree that the clinical learning environment should favor resident autonomy and that the current environment does not achieve the ideal balance. There are several factors contributing to autonomy and supervision, including attending-, resident-, patient-, interpersonal-, and institutional-related. These factors are complex, multifaceted, and dynamic. Cultural shifts towards supervision by primarily hospitalist attendings and increased attending accountability for patient safety and systems improvement outcomes further impacts trainee autonomy.

## Background

Balancing autonomy and supervision during medical residency is important for promoting trainee development while also ensuring patient safety. Several regulatory changes and the evolution of patient safety and quality improvement movements in healthcare over the past twenty years have emphasized increasing the quality of trainee supervision by attending supervisors.^1–3^

Growing tension between trainees and attendings around autonomy and supervision has been reported, particularly when the balance is skewed in one direction or another. Studies have noted that trainees desire more autonomy than attendings are willing to provide, and that attending support for trainee autonomy is perceived to be limited.^4,5^ Both extremes are perceived negatively – too much autonomy can cause feelings of abandonment and hinder trainee’s clinical decision-making, while too much oversight can foster feelings of distrust and prevent trainees from expressing concerns on rounds. Attendings grapple with this balance as their role has expanded on the team in response to pressure to achieve hospital quality metrics or to compensate for team discontinuity related to duty hour restrictions or other residency curricular activities.^6^ For trainees, lack of autonomy has been reported to negatively impact well-being.^7,8^

Appropriate supervision promotes safe patient care, resident development and education, and opportunities for evaluation.^1^ However, there is debate over whether these goals are being achieved. While one meta-analysis noted a slight decrease in patient mortality after the implementation of supervision and duty hour standards, this has not been reproduced in other studies.^9,10^ Similarly, the impact of increased supervision on medical error rates has had conflicting results.^11,12^ Effect on trainee development is also unclear; some studies suggest increased supervision improved the clinical-learning environment, while others found that structured independence positively affected their development, sense of “ownership” over patient care, and overall satisfaction.^13,14^

Entrustment and the variables that affect a supervisor’s confidence in a trainee has been an area of robust research. The basis of entrustment decision-making is a supervisor’s assessment of a learner’s ability, integrity, reliability, and humility; it is specific to a given patient care activity and clinical situation.^15,16^ In an attempt to characterize a trainee’s progress through stages of responsibility toward independent practice, Entrustable Professional Activities (EPAs) have been created to capture behaviors and activities that represent a trainee’s development through residency.^17^ However, these assessments are performed retrospectively by supervising faculty after a period of direct observation, are specific to a single skilled behavior, and are not easily accessible to future supervisors.^18^

In this study, we defined “autonomy” as *the ability to provide patient care in an independent and competent manner* and “supervision” as *the provision of both direct and indirect oversight by a more experienced individual in a clinical learning environment to achieve the aforementioned goals*. This study aimed to understand the current and ideal states of autonomy and supervision with the hypothesis that a discrepancy exists, and further describe the factors that contribute to tension from the perspectives of both trainees and attendings.

## Methods

### StudyDesign

We conducted a mixed-methods study using surveys and focus groups to explore attending and trainee perspectives on autonomy and supervision during inpatient medicine teaching rotations.

### Setting, Participants and Oversight

This study took place at the three teaching hospitals of the University of California-San Francisco (UCSF): quaternary care hospital, public county hospital, and Veterans Affairs (VA) hospital. Eligible participants included internal medicine trainees (interns and residents) and attending physicians from each site.

### Survey development and data collection

We developed two surveys to explore perspectives and practices related to autonomy and supervision during inpatient medicine rotations from trainees and attendings (Appendix 1 and 2). These surveys utilized both fixed-response questions (i.e. 5-point Likert scale) and open-ended free text responses to evaluate perceptions of “current” and “ideal” state of autonomy and supervision, experience with autonomy and supervision during training, perceived value of attending contributions to patient care and medical education, and perceived impact of autonomy and supervision on burnout and wellness. Both surveys presented six clinical scenarios and prompted participants to select their preferred response of autonomy or supervision along a spectrum of behaviors for each scenario. Between May 2019 and June 2020, surveys were distributed to participants using online survey software (Qualtrics, Provo, UT).

### Focus group development and data collection

We developed a study-specific focus group guide informed by survey responses (Appendix 3). Questions were open-ended and followed up with prompts to elicit greater detail about participant’s definitions of autonomy and supervision, contributing factors, impact on work satisfaction, and perception of readiness for independent practice. Participants were invited by email to take part in focus groups between October 2020 and April 2021. Trainee and attending focus groups were held separately; all focus groups were digitally recorded.

### Survey and focus group analysis

Survey responses were summarized using descriptive statistics. Trainee and attending responses were compared using chi-square tests or Fisher’s exact tests. Analysis was completed using SAS 9.4 (SAS Institute Inc., Cary, NC, USA). Survey questions that were open-ended were analyzed using thematic analysis.^19^ At least two members of the research team independently performed open coding of transcripts using a data driven (inductive) approach. To ensure methodological rigor, the research team met at regular intervals to develop a code book and to resolve any coding discrepancies using negotiated consensus.^20^ Codes were then grouped into higher order themes. Focus group data were professionally transcribed, verified for accuracy, and de-identified to ensure confidentiality and limit analytic bias.^21^ We used thematic analysis to summarize focus group data.

## Results

Surveys were sent to 182 trainees (63 PGY1s, 64 PGY2s, 55 PGY3s) and 208 attendings across the three inpatient sites. Seventy-six (42%) trainees and 101 (49%) attendings completed the survey (Table 1a). Fourteen trainees (8%) and 32 attendings (32%) participated in focus groups (Table 1b).

### Survey Results

Across all clinical sites, trainees perceived the current culture to be significantly more autonomous than attendings (Table 2). However, both attendings and trainees described an “ideal” culture as more autonomous than the current state, though trainees favored more autonomy than attendings. The largest difference between current and ideal state occurred at the quaternary referral center, which was perceived to be highly supervisory by both groups.

**Figure 1:**
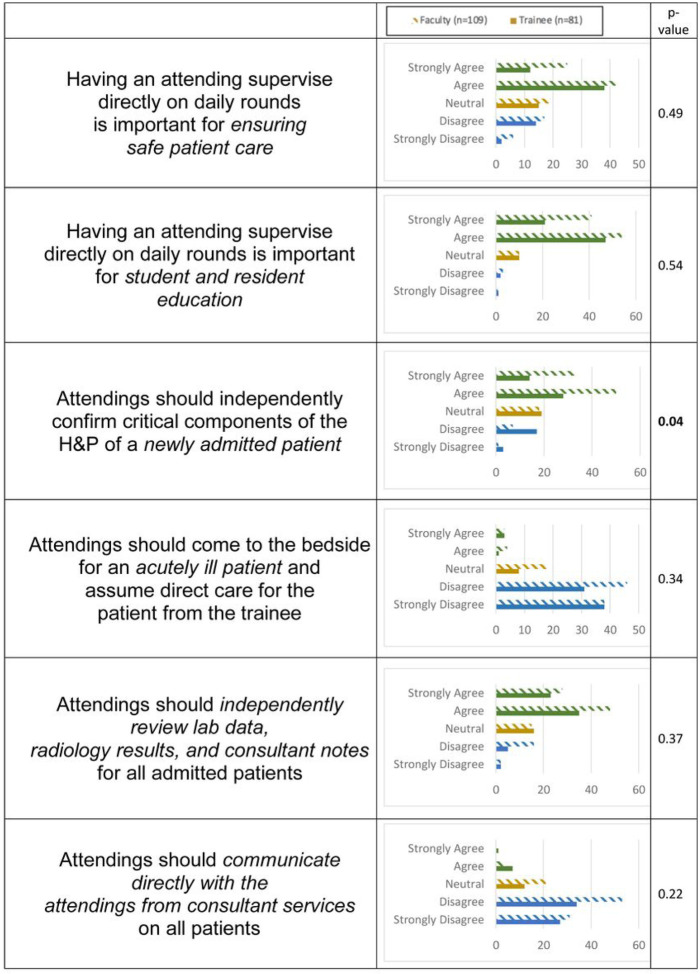
Comparison of attending (n=109, hashed lines) and trainee (n=81, solid lines) responses to attending contributions to various aspects of rounds by 5-point Likert scale. Attendings and trainees generally agreed on the attending’s role in patient safety, education, acute decompensations, review of EHR data, and consultant communication. Attendings perceived a greater need for supervision in newly admitted patients than residents did (p=0.04).

Figure 1 summarizes attending and trainee perspectives of attending contributions to rounds by survey responds. There was good agreement between both groups that attending presence on rounds improved patient safety and trainee education on rounds, resident autonomy was important during the assessment and management of acutely ill patients and during consultant communication, and attendings should review necessary data independently in the Electronic Health Record (EHR). However, attendings valued a more supervisory role in new patient encounters, while trainees expected more autonomy (p = 0.04). Five themes arose from analysis of the six clinical scenarios and open-ended survey questions as core contributors to the autonomy-supervision dynamic: attending-, resident-, patient-, interpersonal-, and institutional-related factors (Appendix 4). Identifying these themes informed development of subsequent focus group questions.

### Focus Group Results

**Figure 2:**
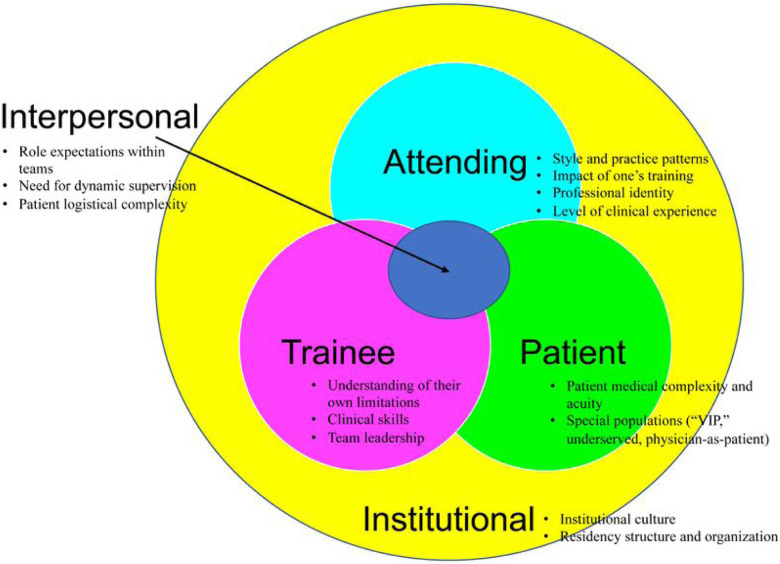
Schematic of the inter-relationship between the five core contributors to the autonomy and supervision balance on inpatient medicine teaching services. Each core contributor (attending-, trainee-, patient-, interpersonal-, and institutional-related factor) had several key subthemes.

Focus group analysis of the five core contributors to the balance of autonomy and supervision revealed a complicated dynamic. We identified subthemes within each core contributor to better understand the multiple variables that influence the balance of autonomy and supervision in the inpatient clinical learning environment (Fig. 2). Representative quotes for each subtheme are summarized in Table 3.

### Attending Contributions

The attending contribution to the autonomy and supervision dynamic was multifaceted, incorporating their practice patterns, reflections from their residency training, and experience navigating the various, and often changing, roles that they are expected to fulfill within the team and institution. There was a near universal intention of restraint in order to create space for trainee clinical decision-making, though execution varied significantly depending on attending risk tolerance for trainee mistakes and flexibility in creating patient care plans. Trainees often were unable to distinguish discomfort with patient-related factors from discomfort with their own clinical skills, which seemed to exacerbate a negative reaction to perceived hypervigilance or “inappropriately” supervisory behaviors and amplify benefits of attendings’ intentional flexibility in clinical decision-making.

Attendings’ perspectives on the value of autonomy and supervision often related to experiences from their own residency training. Many attendings voiced support for resident autonomy due to perceived benefits to their own clinical growth from autonomous practice in residency or were able to identify supportive supervisory behaviors that helped ease workload or navigate specific challenges during their training. In general, attendings were motivated to emulate positive experiences and avoid distressing ones (most often related to inadequate supervision) as a guide to how they approached autonomy and supervision in their own practice.

Attendings described supervising inpatient resident teams as increasingly challenging, with a multifaceted and dynamic role that required adjustments even over the course of one rotation with a given team. They described feeling pressure to ensure high-quality patient care while also satisfying roles as an educator, supervisor, role model, and mentor. Many attendings and trainees identified that expectations for engagement with trainees varied by gender, level of clinical experience, and primary specialty (hospitalist versus subspecialist or outpatient physician). Female attendings expressed more difficulty earning respect and trust from trainees than male counterparts, particularly when their resident was male. Universally, attendings and trainees reported that junior attendings tended to behave or be perceived as more “hands-on” and engaged than senior attendings. This behavior was hypothesized to be related to clinical uncertainty or anxiety, though many attendings cited perceived expectations from trainees as the reason. Trainees were less likely to identify level of clinical experience as a determinant of engagement, and more likely to identify primary specialty. Trainees reported experiencing greater autonomy with subspecialty and outpatient attendings, though this was not necessarily perceived as positive or intentional for their educational or patient care experiences.

### Trainee Contributions

Generally, trainees expected significant autonomy in clinical decision-making, but they appreciated attendings who were responsive to their needs for support or guidance. Few trainees described negative experiences with autonomy; most examples of discomfort occurred from perceived overwhelming or inappropriate supervision.

Both groups highlighted trainees’ expressed understanding of their own limitations (humility, communicating uncertainty, and openness to feedback) as a behavior that promoted autonomy. Participants agreed that self-assessment of one’s own comfort level with patient acuity and complexity, utilizing other team members for their expertise and guidance, and being receptive to other perspectives were critical to developing trust and fostered further autonomy.

Additionally, both groups valued a trainee’s ability to articulate their clinical reasoning and prioritize strong communication amongst the patient and provider team. This was particularly reflected in their ability to describe their thought process on rounds, give insightful and accurate feedback to interns and medical students, and proactively and collaboratively communicate with interprofessional team members.

Lastly, team leadership was identified as an important determinant of autonomy for both attendings and trainees. Residents described that their attentiveness to detail often translated to attendings offering more autonomy, and that interns’ attentiveness to task completion often informed their own decisions about autonomy. Attendings mirrored this statement, noting that a resident’s ability to effectively manage the team, pay attention to the details of inpatient care, and capture important details from the EHR were vital to establishing trust.

### Patient Contributions

There was almost uniform agreement between trainees and attendings that attending supervision was appropriate in the context of high acuity and complex patients, particularly with co-existing clinical uncertainty. Trainees expressed appreciation and feelings of safety when they knew that attendings would support them and engage in clinical decision-making; attendings acknowledged the opportunity they had to empower resident learning and development, while also ensuring patient safety, in these contexts.

Both trainees and attendings described a generational shift regarding the care of at-risk and under-resourced patient populations. Trainees noted that historically, patients who were affected by social drivers of health or without robust support systems were often those with whom they experienced the most autonomy; attendings reported similar experiences during their own training. However, trainees noted that junior attendings and hospitalists now take on a larger role in the care of these patients. Attendings expressed strong feelings of responsibility and desire to help trainees navigate the complex healthcare system to promote improved outcomes for vulnerable patient populations.

Interestingly, patients with privilege and physician-patients were described as cared for holistically and collaboratively by the medical team, with both trainees and attendings citing patient and family expectations, professional courtesy, and streamlined communication as reasons for adjusting their behavior towards these patients.

### Interpersonal Contributions

In addition to any individual team member characteristics or behaviors, the way in which team members interacted and communicated with one another, and how that interaction changed over the course of a shared rotation, was highlighted as a distinct contributor to the autonomy and supervision dynamic. Just as an attending’s practice patterns often informed their approach to the attending role on the inpatient team, trainees had expectations for their own responsibilities. When there was overlap in these roles, strong communication ensured that team members felt valued and supported, rather than hindered and intruded upon. The most frequently described example of this overlap was “task sharing:” when an attending attempted to absorb tasks from the trainee. Attendings described task sharing with trainees when they felt they could be particularly helpful - either because of a specific relationship with a patient and family that had formed, or because of perceived busyness of the trainee - or when they felt that team discontinuity put the attending in a position to fill multiple roles for several days in a row. Trainees had discordant opinions about task sharing; some expressed appreciation for attending willingness to step in, while others found it intrusive on trainee autonomy and growth. However, even with that discordance, both attendings and trainees shared that the impact on team dynamics was improved when expectation setting occurred at the beginning of a rotation and when there was proactive communication about task sharing and roles throughout the rotation.

Both groups identified the benefits and importance of “dynamic supervision” based on the composition of the team that day (accounting for trainee days off). Many attendings described the perceived need to “scale up” supportive efforts during trainee days off, and that these experiences sometimes led them to feel more ownership over patient care than trainees. Trainees, while they acknowledged the attending’s dynamic role, strongly preferred that attendings “scale down” their supervision when the team was whole to allow the resident to assume a team leadership role. Attendings expressed difficulty adjusting levels of supervision when they perceived their role to facilitate continuity for patient care, though residents struggled with their role on the team when attendings did not achieve appropriate dynamism.

Lastly, there were discordant opinions between and amongst both groups over appropriate balance of autonomy and supervision in the context of patients with significant logistical complexity (e.g. multiple consultants contributing to patient care, complicated transitions of care, or previous difficult interactions between a patient and members of the care team). Some trainees and attendings highlighted the benefits of attending involvement in these areas, including their ability to streamline communication between services, navigate a complex healthcare system, and model effective de-escalation and trust-building techniques. Conversely, many communicated concern about attendings and trainees over-prioritizing the ease of having an attending assume responsibility for these fairly common challenges in the inpatient environment at the expense of trainee development.

### Institutional Contributions

These complex interpersonal interactions take place within an institutional context that informed the autonomy and supervision dynamic on the inpatient medicine service, specifically the “culture” of the institution and residency expectations for trainees and attendings. Culture was difficult for participants to define but was described as tightly wound with the “hidden curriculum” of an institution and visible within the team dynamic. Both attendings and trainees noted that they mostly learned their roles by shadowing or experiencing them through their own training (if attendings trained at the same institution), thereby perpetuating whatever culture they had experienced previously. Conversely, new attendings described altering their practice in order to assimilate to the perceived values and expectations of their current peers and trainees. This seemed to act as a balancing measure for the impact of an attending’s own training experience.

The residency program’s organizational structure of the inpatient medicine rotation was noted to impact the balance of autonomy and supervision most prominently by contributing to team discontinuity due to call cycles and trainee days off. Both groups acknowledged that many of these changes were necessary to satisfy ACGME requirements and important to promoting trainee well-being. However, they also negatively impacted the functioning of the inpatient team. In fact, many of the issues mentioned above - need for dynamic supervision, changing expectations of roles within teams, and prioritization of continuity for patient care – were attributed to or worsened by team discontinuity. Both attendings and trainees expressed similar dissatisfaction with a team structure that was often fractured.

## Discussion

In this study, we established that the current climate of autonomy and supervision was perceived as more supervisory than desired by both attendings and trainees. There was generally good agreement on the level of autonomy and supervision that was appropriate across both educational and patient care domains, except when admitting a new patient to the hospital. Subsequent focus groups were able to describe specific features of attendings, trainees, and patients that contributed to the complex dynamic of autonomy and supervision, and how these groups are affected by interpersonal dynamics and institutional factors.

While there has been active research in trainee autonomy and supervision over the past decade, this study is novel in exploring the interactions of factors amongst groups, highlighting previously undescribed factors, and noting a generational shift in how the modern inpatient environment impacts these factors.

Entrustment has long been described as a contributing factor to resident autonomy, though is classically unidirectional - that is, assessed and provided from the direction of the supervisor toward the trainee.^22,23^ However, in this study we found evidence of *bidirectional* entrustment, with trust being applied from trainees towards their attendings for supervisory behaviors, which is a novel understanding of this term. For example, trainees’ assessment of an attendings’ supervision or provision of autonomy in multiple domains influenced how they developed trust in their attending: whether their behaviors were intentional or incidental based on their level of clinical experience, reactive to mistrust of the trainee, reflective of clinical uncertainty, or accommodative to the dynamic supervision necessary over the course of the rotation. This phenomenon of “bidirectional entrustment” has important implications in the modern clinical learning environment where attendings and trainees work in increased partnership rather than in a traditional hierarchy and rely on each other more to achieve patient care and educational goals.

This study described how team discontinuity as a result of residency’s organizational structure has contributed to the erosion of the resident leadership role, and the challenge it creates for the attending to participate in “dynamic supervision.” While team leadership is an important skill in the domain of “Interpersonal and Communication Skills” of the ACGME Core Competencies,^24^ it has become more difficult for residents to meaningfully occupy this role when they may miss one or more days per week due to scheduled days off, post-call days (from overnight call), and other competing residency requirements. Some of this can be mitigated on the residency level by minimizing scheduling conflicts with inpatient rotations, but residency programs should consider the impact of the organizational structure on trainees and their ability to contribute to the inpatient team when balancing this with other priorities. Attendings can and should continue to participate in dynamic supervision, but the numerous challenges identified in this study suggest that this is a stopgap measure to ensure that the resident has an opportunity to occupy a leadership role on the inpatient team, rather than a solution to the problem of team discontinuity.

Prior to this study, the importance of the multifaceted attending identity on the balance of autonomy and supervision in the inpatient environment was not well described. Here we highlight how elements of an attendings’ prior training experience, level of clinical experience, gender, primary clinical role, and risk tolerance for resident mistakes all contributed to their supervisory practice. Additionally, we described how trainees and patients variably perceived and interacted with these factors. Acknowledging the complex identities of all team members, including the attending, is important when establishing the team leadership and communication expectations at the start of a shared rotation. Other studies have demonstrated similar gender disparities of female trainee autonomy and female attending sense of respect and value.^25,26^ Particular attention should be paid to the experience of junior female attendings, as the trust and respect of trainees is critical to effective team leadership and ensuring patient safety.

Lastly, this study demonstrated a generational and cultural shift that represents the evolution of the modern clinical learning environment since the regulatory changes of the early 2000’s. Current attendings hold a unique perspective and exposure to both the traditional hierarchy and pedagogy of medicine, as well as the modern patient safety, health systems improvement, and medical education movements of the past twenty years. This was reflected in attendings’ desires to emulate positive experiences from their training, as well as avoidance of negative experiences of feeling inappropriately unsupervised and unintentionally harming patients. This was also evident in how both groups described care for at-risk and under-resourced patient populations, who were historically perceived to be “opportunities” for more autonomous practice by trainees.^27^ The younger generation of attendings – particularly hospitalists – described an intentional effort to add value to trainee education and patient care for at-risk and under-resourced patient populations through supervision and education around systems-based practice that would result in improved advocacy and outcomes.

In general, there has been a shift towards hospitalists as primary educators on inpatient medicine teaching services that has aligned with the trend towards hospitalists as primary providers for inpatients at many institutions, including ours.^28^ Participants noted several benefits of this shift, including more predictability in day-to-day activities like rounds, better adherence to health system improvement efforts, and improved education and role modeling in patients with complex inpatient and transition of care needs. Additionally, trainees expressed more trust in the autonomy that was granted by hospitalists compared to subspecialists or outpatient physicians who rarely attended on inpatient teaching services. Hospitalists were also more likely to err towards inappropriate direct supervision, consultant communication, or task sharing behaviors than their subspecialist or outpatient counterparts, and could readily intrude on resident autonomy if not mindful and restrained in these practices.

This study has a few limitations. First, while we surveyed and conducted focus groups at three distinct inpatient sites serving different patient populations, all sites are affiliated with a single institution. The attending groups are distinct from one another, but the trainees rotate through all three sites. Second, this study was conducted during the height of the COVID-19 pandemic, which we believe negatively impacted recruitment for participation and limited our ability to do in-person outreach at all three sites. We believe that despite these limitations, the results of this study are adequately descriptive and generalizable.

## Conclusions

The balance of autonomy and supervision on the inpatient medicine service is complex, multifaceted, and dynamic. Trainees and attendings agree that the clinical learning environment should generally favor resident autonomy, and that the current environment does not achieve the ideal balance. When considering the factors contributing to this complex dynamic, one must consider the attending, resident, patient, interpersonal, and institutional variables at play. It is also important to recognize that these factors interact with one another: entrustment is bidirectional between attendings and trainees, and team discontinuity is an important disruptor to the preferred model of resident team leadership. Lastly, it is important for team members - including attendings themselves - to acknowledge how their identities contribute to the autonomy and supervision balance, and that the cultural shift towards more hospitalist attendings and increased attending accountability for patient safety and health system outcomes can further impact trainee autonomy on the inpatient medicine service.

## Figures and Tables

**Table 1:** Demographic characteristics of participants in surveys (a) and focus groups (b).

a. Survey participants			
Trainees	n (%)	Attendings	n (%)
*Level of training*	*76 (100)*	*Experience post-training (years)*	*101 (100)*
PGY-1	19 (25)	0–5	38 (38)
PGY-2	27 (36)	6–10	22 (22)
PGY-3	30 (39)	11 +	41 (41)
*Gender*		*Gender*	
Male	35 (46)	Male	45 (45)
Female	41 (54)	Female	55 (54)
Prefer to self-describe	0 (0)	Prefer to self-describe	1 (1)
		*Primary clinical site*	
		Quaternary care hospital	48 (48)
		Public county hospital	26 (26)
		Veterans Affairs hospital	27 (27)
b. Focus Group participants			
*Level of training*	*14 (100)*	*Experience post-training (years)*	*32 (100)*
PGY-1	1 (7)	0–5	12 (38)
PGY-2	7 (50)	6–10	10 (31)
PGY-3	6 (43)	11 +	10 (31)
*Gender*		*Gender*	
Male	5 (36)	Male	15 (47)
Female	9 (64)	Female	17 (53)
Prefer to self-describe	0 (0)	Prefer to self-describe	0 (0)
		*Primary clinical site*	
		Quaternary care hospital	18 (56)
		Public community hospital	3 (10)
		Veterans Affairs hospital	11 (34)

**Table 2:** Comparison of trainee and attending perceptions of current versus ideal culture by training site from entirely autonomous (0) to entirely supervised (1).

Primary Clinical Site	Current culture Mean (SD)			Ideal culture Mean (SD)		
	Trainee (n = 90)	Attending (n = 101)	p-value	Trainee (n = 90)	Attending (n = 101)	p-value
**Quaternary referral center**	0.42 (0.5)	0.77 (0.42)	< 0.001	0.11 (0.32)	0.27 (0.45)	0.02
**County hospital**	0.15 (0.36)	0.48 (0.51)	<0.01		0.30 (0.47)	0.02
**VA hospital**	0.18 (0.39)	0.38 (0.50)	0.04		0.38 (0.50)	0.01

**Table 3:** Representative quotes from focus group participants describing subthemes of core contributors.

Core Contributor	Subtheme	Representative Quote
**Attending**	Style & Practice Patterns	“I do think that autonomy is very important. I do believe you have to make mistakes. The most I’ve learned in residency was from mistakes I’ve made… trying to constantly protect the residents from making a mistake I don’t think is effective.” (attending)
	Impact of Training	“I do remember situations during my training where I did feel overwhelmed overnight or with new admissions…and feeling that tension of not knowing when to reach out to someone for help, and having some moral distress with that and trying to balance doing things on my own versus doing right by patients. I try extra hard to create an environment in which the learners know that they can approach me… because I wouldn’t want them to be in that situation or to have that distress.” (attending)
	Professional Identity	“When you’re an attending, you wear the hat of a clinician first and foremost, hopefully. But then you are a supervisor, a mentor, you’re psycho-social support, and a teacher… You’re trying to do well at the same time. Knowing that you have to be on your A game every day. You’re not allowed to have an off day as an attending.” (attending)
	Clinical Experience	“When an experienced hospitalist is letting me be autonomous in a situation…[l trust] that they know what the boundaries of safety and effectiveness are…I have a harder time accepting autonomy from people who I know don’t necessarily attend as much - specifically because it may not be a skill that is as frequently practiced for others.” (resident)
**Trainee**	Understanding Limitations	“The most important thing is to know when you need help. I find that attendings will give residents a little bit more leeway when they know if they’re out of their depth, or if they’re uncomfortable, they’ll ask for help.” (resident)
	Clinical Skills	“A foundational characteristic’s the ability to communicate, so communication skills with me as the attending, with the nursing staff, with other ancillary staff, and of course with the patent as well. I think that that is just a foundational component I look for in the learner, and if they do well in that domain then typically I feel more comfortable [granting autonomy].” (attending)
	Team Leadership	“Listening to interns present, making mental notes of things that are inaccurate or not addressed yet you’re interested in, residents I feel comfortable with allowing autonomy will hit each of those point by point.” (attending)
**Patient**	Medical Complexity & Acuity	“I actually have had some attendings say to me “I’m very close right now because I’m personally uncomfortable in this situation, not because I don’t trust you.” And it was a hugely impactful moment for me as a trainee…to know that we both feel uncertain, rather than you feel uncertain in me.” (resident)
	Special Populations	“People who are at high risk of [being] vulnerable to poor outcomes, I give a lot more supervision about how you advocate and do things for patients. I feel like people need a lot more education and micromanaging in how we do things properly for very vulnerable populations.” (attending)
**Interpersonal**	Role Expectations	“I just have some days where attendings felt like they were being helpfull but then they would do a task that actually I wanted to do, and it felt like it took a way my autonomy. I think it’s just hard to find a balance…if there’s not good communication around what is helpful, it feels like they are taking away something that is important to my role.” (resident)
	Dynamic Supervision	“The biggest challenge for me in a supervisory role is the ratcheting between the incredibly detail-oriented nature of the intern (when my interns are off) and the supervisory, big-picture needs of the resident when you’re actually supervising. The more times when I am toggling between those two roles, the more difficulty I have as a supervisor giving my trainees autonomy.” (attending)
	Patient Logistical Complexity	“If the patient has a plan that’s being driven a lot by [a] specialty attending, then there’s often these high-level conversations that are happening attending-to-attending that… I find myself just pulling back. I’m not going to make any headway myself with this patient if all the conversations are happening [at the attending level]..” (resident)
**Institutional**	Culture	“I think there’s a real idea of culture like, “Attendings like us supervise residents like this”… there’s a large pressure culturally to be involved as junior faculty because your other junior faculty colleagues are more hands-on, and that’s what has developed the culture for the residents.” (attending)
	Residency Organization	“When we spend very little time together all as a team, the attending ends up being the person with the most continuity in the team. And so, they sometimes have… the most information, and are in the most informed place to help make decisions. But I think that that gives us less autonomy, because we know less about the patients, potentially. And part of that is the discontinuity of the team.” (resident)

## Data Availability

The datasets used and/or analyzed during the current study are available from the corresponding author on reasonable request.
